# Mechanisms Underlying the Analgesic Effect of Moxibustion on Visceral Pain in Irritable Bowel Syndrome: A Review

**DOI:** 10.1155/2014/895914

**Published:** 2014-07-01

**Authors:** Renjia Huang, Jimeng Zhao, Luyi Wu, Chuanzi Dou, Huirong Liu, Zhijun Weng, Yuan Lu, Yin Shi, Xiaomei Wang, Cili Zhou, Huangan Wu

**Affiliations:** ^1^Shanghai University of Traditional Chinese Medicine, Shanghai 201203, China; ^2^Key Laboratory of Acupuncture-Moxibustion and Immunological Effects, Shanghai University of Traditional Chinese Medicine, 650 South Wanping Road, Xuhui District, Shanghai 200030, China

## Abstract

Irritable bowel syndrome (IBS) is a functional bowel disorder that causes recurrent abdominal (visceral) pain. Epidemiological data show that the incidence rate of IBS is as high as 25%. Most of the medications may lead to tolerance, addiction and toxic side effects. Moxibustion is an important component of traditional Chinese medicine and has been used to treat IBS-like abdominal pain for several thousand years in China. As a mild treatment, moxibustion has been widely applied in clinical treatment of visceral pain in IBS. In recent years, it has played an irreplaceable role in alternative medicine. Extensive clinical studies have demonstrated that moxibustion for treatment of visceral pain is simple, convenient, and inexpensive, and it is being accepted by an increasing number of patients. There have not been many studies investigating the analgesic mechanisms of moxibustion. Studies exploring the analgesic mechanisms have mainly focused on visceral hypersensitivity, brain-gut axis neuroendocrine system, and immune system. This paper reviews the latest developments in moxibustion use for treatment of visceral pain in IBS from these perspectives. It also evaluates potential problems in relevant studies on the mechanisms of moxibustion therapy to promote the application of moxibustion in the treatment of IBS.

## 1. Introduction

IBS is characterized by chronic, recurrent abdominal pain accompanied by abdominal discomfort associated with bowel dysfunction. It is regarded as a disease closely related to the brain-gut axis and has a high incidence rate worldwide [1, 2]. Many gastrointestinal diseases can cause visceral pain, and organ resection or targeted treatment usually can alleviate this pain. However, in IBS, this is not the case, and therefore it attracts much clinical interest [3]. IBS is characterized by abdominal pain and discomfort that must fulfill at least two out of three of the following criteria: (1) relieved by defecation; (2) occurrence that is related to changes in stool frequency; (3) occurrence that affects stool characteristics [4]. IBS is a typical functional gastrointestinal disorder (FGID), and its occurrence is unrelated to structural, organ-associated, or metabolic diseases diagnosed using routine examinations in clinical practice [3]. Recent studies on pathogenesis of IBS have shown that visceral hypersensitivity forms one of its important pathophysiological bases. Currently, no drug that effectively targets a particular mechanism has been developed; however, recent clinical and experimental studies have found that moxibustion in TCM demonstrates a unique functional efficacy in the treatment of visceral pain [5, 6]. Therefore, it is necessary to elucidate the mechanisms underlying the analgesic effect of moxibustion on visceral pain in IBS. The effect of moxibustion and acupuncture on particular points on the body surface, transforming physical stimulation into biological stimulation, the release of active substances in localized regions. This leads to cascade reactions to moxibustion and overall functional regulation. Increased sensitivity in the visceral pain pathways causes a range of gastrointestinal diseases leading to clinical symptoms, and the specific substances that play regulatory roles consist of various gastrointestinal hormones and neurotransmitters. Depending on the location of the analgesic effect of moxibustion on visceral pain in IBS, its mechanisms can be divided into peripheral and central analgesic mechanisms.

## 2. IBS and Visceral Pain

**Figure 1:**
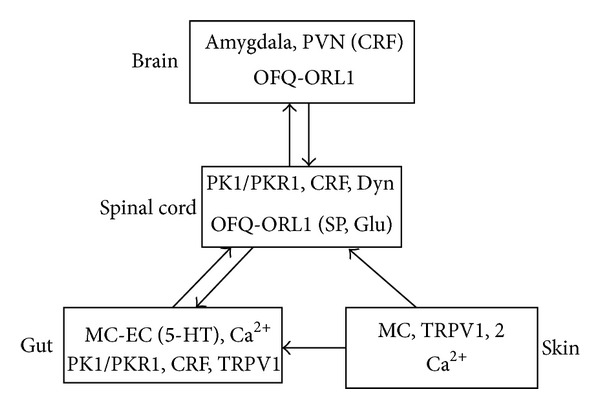
Current known peripheral and central pathways and transmitters associated with moxibustion treatment of visceral pain in IBS.

Visceral hyperalgesia refers to a reduced visceral sensory threshold to normal physiological or noxious stimulations and hence enhanced responses. Currently, it is believed that the pathogenesis of IBS may involve gastrointestinal motility disorders, visceral hypersensitivity, inflammation, brain-gut axis, and mental disorders; however, the specific pathological mechanism of IBS is unclear. The brain-gut interactions play an important role in most pain-related functional gastrointestinal disorders (especially IBS) [7–10]. Known mechanisms of visceral pain pathogenesis include the following: (1) peripheral sensitization: inflammation, injury, or noxious stimuli in peripheral tissues cause sensitization of afferent nerve fibers; (2) central sensitization: sustained, amplified incoming noxious signals from the peripheral are transmitted through the visceral afferent fibers to activate neurons in the spinal dorsal horn; (3) noxious stimulation is transmitted to the spinal cord, leading to activation of endogenous descending facilitation, enhancing transmission of nociceptive information in the spinal dorsal horn. Signals arising from the gastrointestinal tract are transmitted into the brain through the visceral afferent pathways, which can be divided into the parasympathetic and sympathetic afferent pathways [7, 9]. The parasympathetic afferent pathways transmit incoming signals along the vagus nerve to the solitary nucleus, which then transmits the signals to various cortical limbic structures [8]. Sympathetic afferent pathways converge in the dorsal root ganglia and connect to the secondary sensory neurons in layer I of the spinal dorsal horn. The visceral afferent signals are transmitted along the spinothalamic tract to the thalamus and then to insula, cingulate gyrus, and other neuromatrices of the pain. These physiological and pathological mechanisms are also important when studying the mechanisms underlying the analgesic effect of moxibustion on visceral pain in IBS, and they provide references for exploring the mechanisms of moxibustion from the perspective of TCM. Current known peripheral and central pathways and transmitters associated with moxibustion treatment of visceral pain in IBS are shown in Figure 1.

## 3. The Peripheral Mechanism of Moxibustion in Treating Visceral Pain Caused by IBS

Recent studies have demonstrated that visceral hyperalgesia is one of the important pathophysiological bases for IBS. Its mechanism is very complex. Previous studies on its pathological mechanisms have focused on local lesions in the gastrointestinal tract, such as activation of enterochromaffin cells (EC) and mast cells, and abnormal expressions of ghrelin and its receptor in the intestinal tract. Studies on the peripheral mechanism of the analgesic effect of moxibustion on visceral pain in IBS have mainly focused on the cellular and molecular levels, and specific pathways associated with moxibustion treatment of visceral pain in IBS have not been found.

### 3.1. Mast Cells, 5-Hydroxytryptamine (5-HT), and Moxibustion Treatment of Visceral Pain in IBS

Under the regulation of the central nervous system, intestinal sensation and movement are regulated by the intrinsic enteric nervous system and hormones. The enteric nervous system is a relatively independent nervous system that controls neuromuscular function in the gastrointestinal tract. In the terminal ileum, ascending colon and rectum of IBS patients, the number of mast cells, and the degree of infiltration and degranulation are significantly higher than in healthy people [11, 12]. After stress, the mast cell activity is significantly enhanced. They release large amounts of biological active substances to affect the intestinal muscle movement and stomach colon reflection, causing intestinal motility disorders, visceral pain, and other symptoms of IBS. Mast cells are the device that amplifies the response to moxibustion [13]. Enhanced concentration of histamine is the physiological basis of the amplifying effect of mast cells. Moxibustion can activate mast cells enriched in the acupuncture points and promote the mast cells to degranulate and produce bioactive substances that increase the permeability of the capillaries. This accelerates the flow of tissue fluid, further activates mast cells, and allows the secretory products of the mast cells to spread to distant locations with the tissue fluid, thereby activating mast cells and neuronal cells distributed along the meridian lines, and conducting the stimulation effect of moxibustion on acupuncture points along the meridians [14, 15]. It has been reported that in rat models of IBS visceral pain, the number of mast cells in the skins at the Tianshu point is significantly increased compared to control rats, and there is notable degranulation. Moxibustion can increase the number, distribution area, and degranulation of mast cells at the Tianshu point [14], thereby treating visceral pain in IBS. In addition, the degranulation of mast cells is enhanced with increased temperature of moxibustion within a certain range [16]. Moxibustion can also significantly reduce the number of mast cells in the colon of IBS rat models [17].

ECs are distributed throughout the gastrointestinal mucosa, generating approximately 90% of all 5-HT in the gastrointestinal tract. The remaining 5-HT is secreted by the enteric nervous system. 5-HT is a brain-gut peptide widely distributed in the central nervous system and the gastrointestinal tract, and it is an important neurotransmitter regulating the gastrointestinal function. Changes in the 5-HT system are closely associated with abdominal pain, diarrhea, abnormal visceral sensation, and other symptoms in IBS patients, and the 5-HT system is an important signaling system for maintaining intestinal balance. A clinical study on moxibustion treatment of visceral pain in IBS has shown that 5-HT expression in the colon mucosa of IBS patients is significantly increased compared to normal human, and moxibustion can regulate the changes in 5-HT levels in the colon mucosa of IBS patients; that is, moxibustion can improve IBS symptoms and downregulate 5-HT expression [18]. In addition, moxibustion can also lower the 5-HT concentration in the plasma of IBS patients [19]. By reducing 5-HT in colon mucosa and plasma, moxibustion reduces the sensitivity of the enteric nervous system and improves the gastrointestinal motility, endocrine function, and visceral hypersensitivity in IBS patients [20]. Animal studies have also confirmed that moxibustion can adjust the 5-HT pathway in the colon to reduce the degree of visceral pain in IBS rats [21, 22], which is similar to the mechanism of electroacupuncture [23, 24]. The involvement of mast cells from peripheral acupuncture points to the colon suggests that mast cells and 5-HT released by mast cells mediate the peripheral mechanism of the analgesic effect of moxibustion on visceral pain in IBS.

### 3.2. Prokineticin 1

Prokineticin 1 (PK1) is a newly discovered endogenous regulator of gastrointestinal motility. PK1 and prokineticin receptor 1 (PKR1) also mediate signal transduction in visceral hypersensitivity by participating in the regulation of gastrointestinal motility in IBS rats [25]; PKR1 knockout rats show Bv8-mediated hyperalgesia, weakened response to noxious heat stimuli, and reduced sensitivity to heat and mechanical stimulation [25]. Moxibustion can regulate the expression of PK1/PKR1 in the colon of rats with chronic visceral hypersensitivity [26]. The involvement of PK1 and its receptor in the peripheral mechanism of the analgesic effect of moxibustion on visceral pain in IBS has been confirmed in the colon [26]. Further studies on the central mechanism have found that at the Tianshu point (ST25) moxibustion can also reduce the expression of PK1 and PKR1 in the spinal cord of IBS rats [27], thereby inhibiting the transmission of pain signals as a result of IBS visceral hypersensitivity to the spinal cord dorsal horn neurons and higher areas in the central nervous system.

### 3.3. TRP Channels

The capsaicin receptor belongs to the transient receptor potential superfamily of ion channels. The immunoreactive transient receptor potential cation channel subfamily V member 1 (TRPV1) value in the nerves fibers in the rectosigmoid colon of IBS patients is 3.5 times that of normal human. TRPV1 expression is significantly correlated with the pain severity, and TRPV2 shows a strong mechanical sensitivity [28]. In the preparation of a rat model of IBS visceral hypersensitivity, the applied colorectal stimulation is a mechanical stimulation. This stimulation may be sensed by TRPV2, which is therefore involved in the generation of IBS visceral hypersensitivity. Moxibustion temperature can change the local temperature at the acupuncture points, thereby affecting the expression and function of TRP proteins sensitive to thermal stimulation. In a rat model of IBS visceral hypersensitivity, high gene and protein expressions of TRPV1 and TRPV2 are found locally in the Tianshu point, and moxibustion can effectively downregulate the expressions of TRPV1 and TRPV2 mRNA and proteins [29]. TRPVR1 expression in the acupuncture point area is associated with the heat stimulation in moxibustion, and only when temperature >43°C will TRPVR1 be activated. Moxibustion causes a mild thermal stimulation. When the local temperature is higher than 43°C, TRPVR1 in the moxibustion area is bound to be activated, thereby opening cation channels in the cell membrane and cation inflow (especially Na^2+^ and Ca^2+^) from outside the cell into the cell. This generates action potentials, which transmit the moxibustion stimulation via nociceptors in the nerve fibers to the central nervous system, thereby playing an analgesic effect [30].

### 3.4. Calcium Ions

The mild thermal stimulation of moxibustion can be captured by the thermoreceptors on the skin and then activate related proteins in the TRP family. TRP family members are nonselective cation channels with high permeability for Ca^2+^. The members of the TRP family distributed on mast cells are mainly TRPV1 and TRPV2, which are present in their cell membrane or intracellular organelle membrane and can cause a large amount of Ca^2+^ influx when activated [31, 32]. Ca^2+^ serves as an intracellular messenger, and changes in its concentrations will induce a series of cascade biological events [33]. At meridian points Ca^2+^ is enriched locally [34], and the effect of moxibustion on prevention and treatment of diseases via the meridian points is bound to affect Ca^2+^ concentration. In the formation of IBS visceral hypersensitivity, Ca^2+^ may play an important role. It is transferred in the body, reduced in the meridian points on the body surface, and enriched in the intestine [35]. Calcium antagonist (pinaverium) is effective in treating IBS. By inhibiting Ca^2+^ influx and the induced mast cell degranulation, it reduces neurotransmitter release from mast cells in the colon, thereby alleviating abdominal pain and discomfort in IBS patients. Moxibustion at the Tianshu point can cause Ca^2+^ redistribution in the point area. As the temperature of moxibustion increases, Ca^2+^ concentration in mast cells also increases accordingly [29], yet the Ca^2+^ concentration in the intestine may decrease. This also plays a role in improving IBS visceral pain.

## 4. The Central Mechanism of Moxibustion in Treating Visceral Pain Caused by IBS

The mechanism of visceral hyperalgesia is very complex and involves both the peripheral nervous system and all levels of the central nervous system [30]. Recent studies have found that central sensitization is a key factor in the development of visceral pain. It has been reported that inhibition of central sensitization can effectively relieve chronic visceral pain [36]. Abnormal elevation in the excitability of visceral sensory neurons and interneurons in the dorsal horn is an important mechanism of chronic visceral hyperalgesia in IBS. Multiple neurotransmitter and receptor systems are involved in the central sensitization in the spinal cord [37, 38]. Mechanisms underlying the involvement of some of the neurotransmitters related to the analgesic effect of moxibustion on visceral pain in IBS have been revealed, although the central mechanisms of moxibustion in pain alleviation still need further investigation. This part of the review will help to guide future studies.

### 4.1. Corticotropin-Releasing Factor (CRF) and Its Receptor System

Stress from intestinal irritation or psychological origin can activate the hypothalamic-pituitary-adrenal (HPA) axis. Neurons located in the paraventricular nucleus and amygdala of the hypothalamus rhythmically secrete CRF to control the rhythm of the entire system. Various factors, including genetic or acquired, may cause reactive changes in HPA-CRF and affect the perception of visceral stimuli. Moxibustion can downregulate the CRF level in the hypothalamus of IBS rats with visceral pain and reduce their visceral pain sensitivity [39]. CRF promotes 5-HT release in the periphery. As previously described, 5-HT is involved in the regulatory effect of moxibustion on colonic motility and visceral sensation together with CRF. Under noxious stimulation, stress induces overexpression of CRF in the peripheral colon and spinal cord, and CRF then binds to its receptors CRFR1 and CRFR2. The activation of the CRF-CRFR1 signaling pathway leads to visceral hyperalgesia, whereas the activation of the CRF-CRFR2 signaling pathway relieves visceral hyperalgesia. In IBS-like chronic visceral hyperalgesia, the CRF-CRFR1 signaling pathway predominates. Studies at the peripheral colon and spinal levels have shown that moxibustion can reduce the gene and protein expressions of CRF and CRFR1 while increasing the gene and protein expression of CRFR2 in rat colon and the corresponding spinal segments. Combined with behavioral results, it has been found that the reduction of CRF and CRFR1 expression and increase in CRFR2 expression in the colon and the corresponding spinal segments, ultimately restoring the rhythmic balance of the HPA axis system [40], are closely associated with the central mechanism of the analgesic effect of moxibustion on visceral hyperalgesia in IBS rats.

### 4.2. Endogenous Opioid Peptides

The five classes of endogenous opioid peptides (EOP) in the spinal cord are considered classic analgesic substances. They bind to different opioid receptors (*μ*, *δ*, *κ*, opioid receptor-like receptor (ORL1)) in the central nervous system and play a role in analgesia and pain adjustment at different levels. Dynorphin binds to its selective *κ* receptor to play an analgesic role at the spinal level. Fos protein can bind to multiple AP-l transcription factor binding sites on the gene of prodynorphin, which is a precursor of dynorphin, with high affinity, and affect its transcription as a transcription factor, leading to increased expression of dynorphin [41], thereby suggesting that the dynorphin gene may be the target gene of the regulatory effect of Fos. The analgesic effect of moxibustion can be partially reversed by intrathecal application of *κ* receptor antagonist and further strengthened by intrathecal application of dynorphin A1-17 [42]. This suggests that in IBS rats with chronic visceral pain, the dynorphin system at the spinal level is involved in the analgesic effect of moxibustion. In the spinal cord of rat models of IBS visceral pain, the expression levels of preprodynorphin (PPD), dynorphin, and *κ* receptor mRNA are all significantly higher than in normal rats, and after moxibustion treatment, these expressions are further enhanced [43, 44]. This indicates that moxibustion intervention may activate the spinal dynorphin system to alleviate chronic visceral pain. In addition, moxibustion affects enkephalin in the spinal cord of rat with IBS visceral pain in a similar way [43]. After stimulation of receptors in the Shangjuxu and Tianshu points, the moxibustion signals are transmitted to different levels of the central nervous system and project to the same (or adjacent) spinal segments (L6-S1) as colorectal nociceptive signals. Some of the moxibustion signals produce inhibition within the spinal segments, thus affecting ascending transmission of the pain signal. This process involves a variety of EOPs, such as enkephalin, dynorphin, and endorphin [45]. It can be deduced that some moxibustion signals ascend along the dorsolateral funiculus of the signal cord, activating the descending inhibitory system of the endogenous pain modulation system (periaqueductal gray (PAG) serves as the core), which acts on the dorsal horn of the spinal cord. The combined effect of spinal segmental inhibition and descending inhibition of the pain modulation system activates the spinal dynorphin system and shows central analgesic effect of moxibustion via presynaptic and postsynaptic mechanisms.

### 4.3. Orphanin FQ- (OFQ-) ORL1

OFQ in the brain induces hyperalgesia and can antagonize morphine analgesia, whereas spinal OFQ is analgesic and can strengthen morphine analgesia [46]. At the Tianshu and Shangjuxu points, moxibustion promotes spinal OFQ synthesis and ORL1 mRNA expression in IBS rats with visceral hyperalgesia and alleviates chronic visceral hyperalgesia by activating the spinal OFQ-ORL1 receptor system. This effect can be partially blocked by intrathecal application of ORL1 specific antagonists [40] and strengthened by intrathecal injection of OFQ, suggesting that the OFQ-ORL1 receptor system at the spinal cord level is involved in relief of chronic visceral hyperalgesia by moxibustion. It has also been shown that intrathecal injection of OFQ and moxibustion can both reduce the expression of substance P (SP) and Glu in the dorsal horn of visceral hyperalgesia rats. Moxibustion promotes the synthesis and release of endogenous OFQ in L6-S1 of rat models of chronic visceral pain and promotes ORL1 gene transcription, acting on neurons in the dorsal horn of the spinal cord, or primary nociceptive afferent fibers, thereby showing an analgesic effect [40]. This suggests that inhibition of Glu and SP in the spinal dorsal horn is one of the pathways through which OFQ-ORL1 participates in analgesia induced by moxibustion, and the analgesic effect of moxibustion and intrathecal injection of OFQ can act synergistically.

## 5. Conclusion and Future Directions

Although the incidence of IBS and its impact on human life is high, its pathophysiological mechanisms remain unclear. Previous mechanistic studies have found that gastrointestinal motility disorder, visceral hypersensitivity, and the brain-gut axis interaction can all partially explain the pathogenesis of IBS, and the interaction of multiple factors is prominent. Moxibustion has shown satisfactory clinical efficacy, and its medical cost is low. Studies have continued to demonstrate the distinct advantages of moxibustion and provide some reliable evidence for explaining the mechanism of moxibustion treatment of IBS. The application of neurophysiological and functional brain imaging techniques in IBS studies helps to reveal the mechanism underlying the treatment effect of moxibustion on IBS patients with visceral pain at the level of the central nervous system, especially in studies on the mechanisms of IBS associated with emotional factors [47], and promotes in-depth clinical investigations on the pathological mechanisms of human IBS.

Moxibustion therapy acts on meridian points, and the mechanism of its treatment effect on IBS involves a number of organs and targets; however, relevant studies were from different points of view and current systematic and comprehensive researches are still lacking. For example, there have not been studies on specific inhibitors of the treatment effect of moxibustion, or on whether there are specific pathways through which moxibustion acts. Previous studies have focused on the regulation of the target organs. Whereas the effect of moxibustion is realized via stimulation of acupuncture points, the exact pathways through which moxibustion regulates the target organs remain unclear. These issues need to be further addressed. In addition, calcitonin gene-related peptide (CGRP), cholecystokinin, and neurotransmitters also play an important role in IBS visceral hypersensitivity, while central tachykinins (SP, NK1-3), NMDA, purinergic receptors, and other neurotransmitters also play important roles in the central mechanism of visceral hyperalgesia in IBS, and studies on the analgesic mechanisms of moxibustion via these neurotransmitters are yet to be carried out. Currently, there are still no universally accepted IBS animal models to study visceral pain [48]. None of the available models can fully reflect the clinical manifestations and pathogenesis of IBS. An ideal animal model of IBS must integrate all relevant factors, including physical, cognitive, emotional, behavioral, and other factors. Establishing an ideal IBS animal model will help clarify the etiology and pathogenesis of IBS and standardize moxibustion treatment strategies. With the methods and results of modern medical researches, the mechanism of the treatment effect of moxibustion on IBS will be better elucidated in the future.
